# Primary Tubercular Sialadenitis – A Diagnostic Dilemma

**Published:** 2019-01

**Authors:** Nitish Virmani, Jyoti Dabholkar

**Affiliations:** 1 *Department of Otorhinolaryngology, Dr. Baba Saheb Ambedkar Hospital, Delhi, India.*; 2 *Department of Otorhinolaryngology, Seth G.S Medical College and KEM Hospital, Mumbai, India.*

**Keywords:** Parotid gland, Sialadenitis, Tuberculosis, Submandibular gland, Salivary gland calculi, Salivary fistula

## Abstract

**Introduction:**

Involvement of the salivary glands in tuberculosis is rare, even in countries where tuberculosis is endemic. It can occur by systemic dissemination from a distant focus or, less commonly, as primary involvement. This article focuses on its myriad clinical presentations that pose a diagnostic challenge to the clinician. We discuss the schema of investigations required to confirm the diagnosis and the limitations faced in the low-cost setting of a developing country.

**Materials and Methods:**

Medical records, including history, physical examination and imaging findings, and the results of cytological, microbiological and histopathological studies of patients diagnosed with primary tubercular sialadenitis were retrieved and analyzed.

**Results:**

Seven patients were treated over a 2-year period. The most common mode of presentation was a painless mass of the involved gland in four patients. One patient each presented with chronic non-obstructive sialadenitis, sialolithiasis, and acute suppurative sialadenitis. Fine needle aspiration cytology was diagnostic in five out of seven cases (71.4%), while mycobacterial culture was positive in two patients (28.6%). In one patient, a diagnosis could only be reached on histopathological examination of the resected gland.

**Conclusion:**

We recommend cytology studies, acid-fast bacilli staining, and mycobacterial culture as the initial investigation on the aspirate in suspected patients, while polymerase chain reaction should be reserved for negative cases. A high index of suspicion, early diagnosis, and timely institution of anti-tuberculosis treatment is essential for establishing cure. The role of surgery in diagnosed cases of tuberculosis is limited.

## Introduction

Tuberculosis is a necrotizing granulomatous disease with a high morbidity and mortality. Extrapulmonary tuberculosis accounts for at least 30% of tuberculosis cases in India. Involvement of the salivary glands is rare and may occur secondary to systemic dissemination from a distant focus, especially the lungs or, less commonly, as a primary tuberculous sialadenitis. Primary involvement presents with a myriad of clinical features, and therefore a high index of suspicion is necessary to diagnose this entity. Different diagnostic modalities include fine needle aspiration cytology (FNAC), acid-fast staining, mycobacterial culture, and tuberculosis-polymerase chain reaction (TB-PCR). At times, however, excision of the involved gland becomes inevitable and the diagnosis is made post-operatively. A high index of suspicion, early diagnosis, and the timely institution of anti-tuberculosis treatment is essential for establishing a cure.

## Materials and Methods

The study was carried out in the Department of Otorhinolaryngology at King Edward Memorial Hospital, a tertiary care referral center in India. We accessed the clinical records of our department to search for patients diagnosed with primary tuberculosis of the major salivary glands over the past 2 years. Their medical records, including history, physical examination, and imaging findings, and the results of cytological, microbiological, and histopathological studies were retrieved and analyzed. The patients were prospectively followed up to assess for any relapse following completion of their anti-tubercular therapy. Informed consent was obtained for their inclusion into this study. The literature on this entity was also reviewed.

## Results

We found seven patients diagnosed with major salivary gland tuberculosis, four of whom were female and three were male. The average age at presentation was 36.1 years, with the youngest patient being a 10-year-old girl and the oldest being a 76-year-old woman. The submandibular gland was the most common site of involvement (57.1%), followed by the parotid gland (42.8%). Clinical characteristics, imaging findings, and the results of cytological and microbiological studies are shown in Tables 1 and 2.

**Table 1:** Clinical characteristics and investigations in patients with primary tuberculosis of the submandibular gland

	**Clinical characteristics**	**Imaging**	**FNAC**	**Microbiology**	**Histopathology**
1	Chronic obstructive sialadenitis; painful swelling with calculus palpable in proximal duct	USG: Bulky submandibular gland with altered echotexture and few calcific foci	Chronic sialadenitis	Not sent	Necrotizing granulomatous inflammation. Multiple epithelioid granulomas with prominent Langhans giant cells
2	Chronic sialadenitis; Swelling with occasional pain	USG: Diffuse enlargement; non-specific	Necrotizing granulomatous inflammation	Negative	–
3	Recurrent painless mass with fistula formation; history of ATT in past for same		Necrotizing granulomatous inflammation	Positive culture	–
4	Painless mass for 2 months with cervical lymphadenopathy at level 2	USG: Two hypoechoic lesions in submandibular gland; multiple necrotic lymph nodes	Necrotizing granulomatous inflammation	Negative	–

ATT: Anti-tuberculous treatment, FNAC: Fine needle aspiration cytology, USG: Ultrasonogram

**Table 2:** Clinical characteristics and investigations in patients with primary tuberculosis of the parotid gland

	**Clinical characteristics**	**Imaging**	**FNAC**	**Microbiology**	**Histopathology**
1	Painful parotid swelling × 15 days with signs of inflammation; Pus on aspiration	Ultrasound suggestive of parotid abscess	Non-specific inflammation	Positive	–
2	Bilateral painless, diffuse, parotid swellings for 1 month. Right sided grade 3 LMN facial palsy × 10 days	MRI : Bulky parotids, T1 hypointense and T2 hyperintense with heterogeneous contrast enhancement	Necrotizing granulomatous inflammation	Negative	–
3	Painless parotid swelling with multiple non-healing fistulas	CT : Bulky left parotid gland with multiple, necrotic intraparotid lymph nodes	Necrotizing granulomatous inflammation	Negative	–

LMN: Lower motor neuron, MRI: Magnetic resonance imaging, CT: Computed tomography

**Fig 1:**
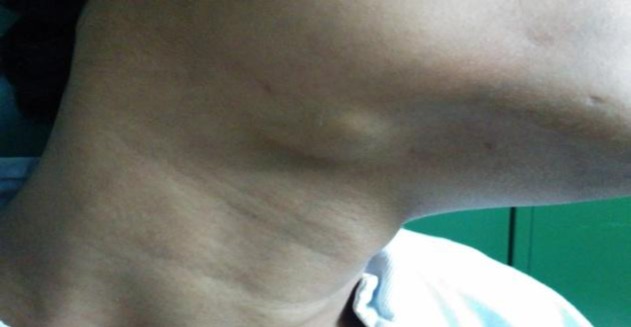
Right submandibular swelling, which increases with food intake. Associated with palpable calculus in submandibular duct

**Fig 2:**
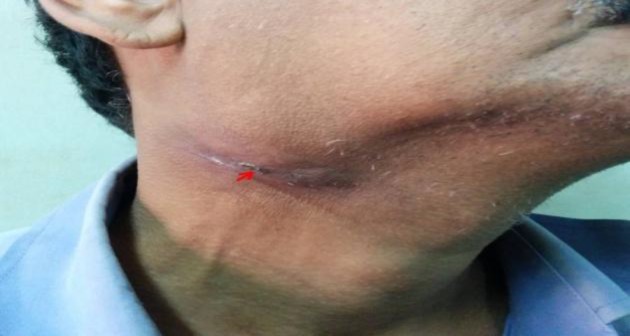
Right submandibular swelling associated with pain. Arrow marks the site of FNAC

**Fig 3:**
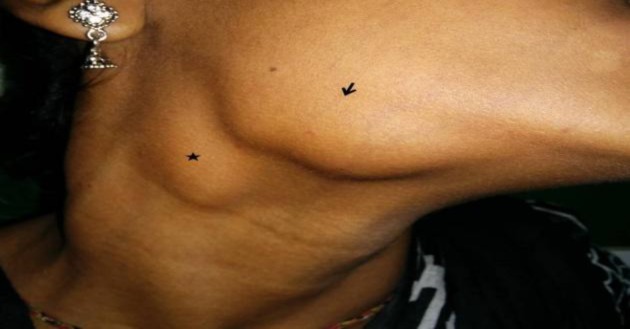
Submandibular swelling (arrow) associated with lymph node enlargement at level 2 (star)

**Fig 4:**
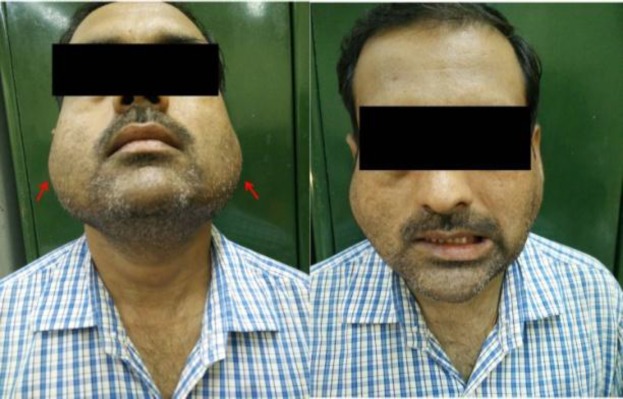
Bilateral diffuse parotid swelling with right sided grade 3 facial palsy

**Fig 5:**
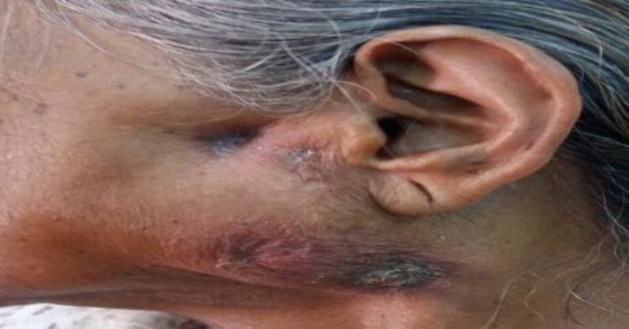
Parotid swelling with multiple fistulas over it

**Fig 6:**
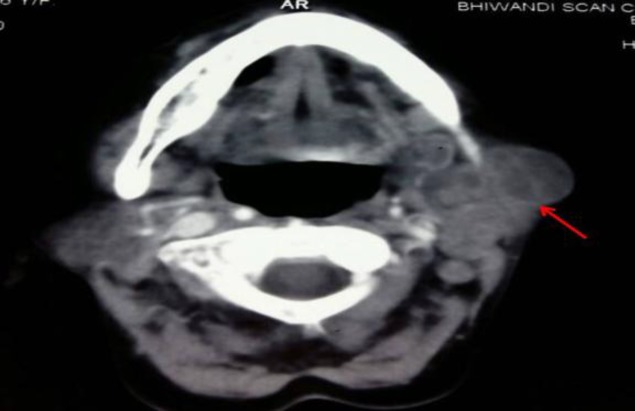
Contrast-enhanced CT scan of previous patient showing a bulky left parotid gland with multiple necrotic intra-parotid lymph nodes

As can be seen, the patients presented with varied clinical features. The most common mode of presentation was a painless mass of the involved gland in four patients. One patient each presented with chronic non-obstructive sialadenitis, sialolithiasis, and acute suppurative sialadenitis (Fig.1-6).

FNAC was diagnostic in five out of seven cases (71.4%), while mycobacterial culture was positive in two patients (28.6%). In one patient, the diagnosis could be reached only on histopathological examination of the resected gland.

**Fig 7:**
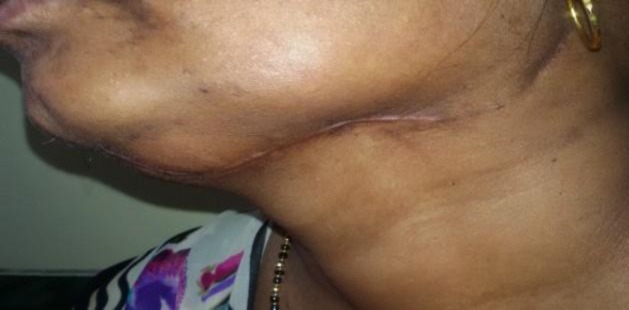
Healed scar post-excision of submandibular gland and fistula

All patients received a complete course of anti-tuberculous drugs. Surgery was performed in two patients. In one case, the diagnosis of tuberculosis was made post-operatively in a patient presenting with classical features of sialolithiasis, including a palpable calculus. In the second, surgery was performed to excise the gland and a non-healing fistula in a case of repeated relapse of submandibular gland tuberculosis (Fig.7).

All patients remained asymptomatic and disease free at 1 year following completion of treatment.

## Discussion

Tuberculosis is a necrotizing granulomatous disease with varied clinical presentations and a wide distribution. Even though the incidence of tuberculosis is on the decline in developed countries, it still exists on a significant scale in developing countries. According to the World Health Organization (WHO) global tuberculosis report (2015), one fourth of global incident TB cases occur annually in India (1). Extrapulmonary tuberculosis accounts for around 30% of tuberculosis cases in India (2). The head and neck region accounts for 10% of extrapulmonary sites, of which the cervical lymph nodes are the most commonly affected, followed by laryngeal tuberculosis, deep neck space abscess, and tuberculous otitis media (3,4). Other less common sites include the skin, oral cavity, oropharynx, nose, thyroid, salivary glands, and mandible. Involvement of the salivary glands is rare, even in countries where tuberculosis in endemic. This could possibly be due to the presence of thiocyanate ions and proteolytic enzymes such as lysozymes in salivary gland secretions. Moreover, the continuous flow of saliva would likely prevent lodging of the bacteria (5).

Two theories can explain the pathogenesis of tuberculous sialadenitis. Most commonly, the involvement of salivary glands occurs secondary to systemic dissemination from a distant focus, especially the lungs, via hematogenous or lymphatic spread. Secondary tuberculosis involves the submandibular and sublingual glands more frequently than the parotid glands. Rarely, an infected source of mycobacteria in the oral cavity, such as tonsils or teeth, liberate these bacilli, which then spread to the nearby salivary gland through direct inoculation by sputum, retrograde ascent of bacilli through the salivary duct or by afferent lymphatics to its associated lymph nodes. This primary tuberculous involvement of the salivary glands is rare and, unlike the secondary form, the most common site of involvement in the literature seems to be the parotid gland (70%) (6). However, in our retrospective review, primary tubercular sialadenitis occurred most commonly in the submandibular gland followed by the parotid gland.

The local spread of the mycobacteria should be more to the submandibular gland, as the opening of Wharton’s duct is present in the dependent part of the oral cavity (i.e., the floor of the mouth). In addition, lymphatic drainage from the oral cavity occurs mainly to the submandibular lymph nodes. Conversely, however, another view holds that the parotid glands may be affected more in localized spread because of the sluggish flow of saliva at rest (7,8).

Tubercular sialadenitis notoriously has a multitude of clinical presentations, often contributing to a diagnostic dilemma. The most common mode of presentation in our review was a slowly growing, painless mass of the involved gland. In this situation, it mimics a neoplasm which is far more common than tubercular sialadenitis. One of our patients had bilateral parotid tuberculosis with facial palsy. Bilateral parotid involvement is very rare, and only a few cases have been reported in the literature (8-10). Differentials include autoimmune parotitis, Sjogren’s syndrome, and sarcoidosis, among others. Facial palsy has rarely been reported, and would tend to present as slowly progressive affection of facial functions (11).

Parotid tuberculosis may present with an abscess, as in one of our cases. However, the clue to the possibility of tuberculosis in this patient was resistance to the usual antibiotics and recollection of fluid after repeated aspiration. Two of our patients presented with chronic gland enlargement associated with pain, thus mimicking chronic sialadenitis. One of them even had a palpable calculus in the submandibular duct, making it impossible for us to even suspect tuberculosis in this case.

Constitutional symptoms such as fever, weight loss, or loss of appetite are usually absent in primary tubercular sialadenitis, and were present in only one of our cases. If present, they are an important pointer toward the possibility of tuberculosis. Since physical examination is often unrewarding in these cases, the burden of diagnosis falls on a high index of suspicion and reliable diagnostic modalities.

FNAC is advocated as a useful and reliable technique for the diagnosis of tubercular sialadenitis (12-14). FNAC is reported to have a sensitivity of 81–100% and a specificity of 94–100% in parotid lesions, and is often the first investigation performed in the evaluation of a parotid or submandibular gland mass (15). We found FNAC to be 71.4% sensitive in detecting tuberculosis of the salivary glands. However, imaging generally does not yield findings diagnostic of tuberculosis, and ultrasonography can increase the yield of aspirate. USG-guided FNAC has the distinct advantage of taking the sample from a more representative area. However, multiple passes may be required to achieve adequate samples for cytological study.

The aspirate can also be sent for acid-fast bacilli (AFB) staining and mycobacterial culture (Lowenstein-Jensen [L-J] medium). However, these conventional methods lack sensitivity for diagnosis of extrapulmonary tuberculosis (28.6% in our series). This could perhaps be due to an unequal distribution of AFB in large volumes of fluids. For the smear to be AFB positive, the sample should contain at least 10,000 bacilli/ml. L-J culture is still considered to be the gold standard, but 10–100 viable bacilli are mandatory for culture positivity (16). However, it is important to note that the culture was positive in a case in which FNAC had failed to detect tuberculosis. Therefore, FNAC should be combined with mycobacterial culture to increase the yield of diagnosis (85.7% in our review). Another added advantage of culture is that it allows for antibiotic susceptibility testing, especially in resistant cases.

The more recently developed PCR is the single most sensitive technique available to date for the demonstration of mycobacterium tuberculosis in the aspirate (17). The advantages of IS6110 PCR are that it is very rapid and easy to perform, and the results can be issued for early treatment and to prevent further transmission of tuberculosis infection. The drawbacks, however, are its false positivity, and its inability to differentiate live and dead organisms (16). Moreover, in developing countries such as India, PCR is limited by its high cost and low availability. Thus, in a low-cost setting, USG-guided FNAC should be performed in conjunction with AFB staining and mycobacterial culture as the initial investigation, while PCR may be reserved for negative cases with a high index of suspicion. At times, however, it may be impossible to diagnose tubercular sialadenitis pre-operatively, and patients undergo excision of the involved gland with a diagnosis made on histopathological examination of the resected specimen. The presence of granuloma with central necrosis surrounded by epithelioid cells and Langhan’s giant cells is the histopatho- logical hallmark of tuberculosis.

Tuberculosis is a medically curable disease. A four-drug regimen (rifampicin, isoniazid, ethambutol, and pyrazinamide) in the intensive phase followed by two drugs (rifampicin and isoniazid) in the continuation phase is the recommended treatment. A complete course of treatment should be given for cases which are diagnosed post-operatively following excision of the gland. The role of surgery in diagnosed cases of tubercular sialadenitis is limited. We performed submandibular gland and fistula excision in one patient with a repeated relapse of submandibular gland tuberculosis, with the rationale to remove the focus of tuberculosis. We prescribed this patient a concurrent course of anti-tuberculous drugs, and she has been relapse-free for over a year now.

## Conclusion

Primary tubercular sialadenitis is a rare entity with a myriad of clinical presentations. A high index of suspicion is a prerequisite, especially in an endemic country like India. We recommend cytology studies, AFB staining, and mycobacterial culture as the initial investigation on the aspirate in suspected patients, while PCR should be reserved for negative cases. At times, however, excision of the involved gland becomes inevitable and the diagnosis is made post-operatively. The role of surgery, however, for diagnosed cases is limited. A high index of suspicion, early diagnosis, and timely institution of anti-tuberculosis treatment is essential for establishing a cure.
